# Endoscopic Management of Calcific Tendinopathy in the Proximal Hamstring: Two Case Reports

**DOI:** 10.1155/cro/5545748

**Published:** 2026-03-01

**Authors:** Hussein A. Elkousy, Davin K. Fertitta, Christopher Fernainy, Sayf Elkousy, Corey F. Hryc

**Affiliations:** ^1^ Fondren Orthopedic Research Institute, Texas Orthopedic Hospital, Houston, Texas, USA; ^2^ Reno School of Medicine, University of Nevada, Reno, Nevada, USA, unlv.edu

**Keywords:** arthroscopic repair, calcific tendinopathy, proximal hamstring

## Abstract

This report presents two cases of previously undocumented proximal hamstring calcific tendinopathy, diagnosed and treated with endoscopic management. A 54‐year‐old woman with a 50% partial‐thickness tear and a 72‐year‐old woman with a 90% tear and knee pathology underwent endoscopic debridement and tendon repair, achieving symptom relief. Neither patient had a preoperative diagnosis of calcific tendinopathy, emphasizing MRI limitations. These cases suggest that calcific tendinopathy of the proximal hamstring may be underrecognized and that endoscopic management offers direct visualization, effective debridement, and minimally invasive repair with low morbidity. Endoscopic evaluation should be considered for patients with persistent hamstring pain despite conservative treatment.

## 1. Introduction

Calcific tendinopathy is a pathological condition marked by calcium deposition, mainly hydroxyapatite crystals, within tendons, most commonly in the rotator cuff tendons of the shoulder [1, 2]. The pathophysiology is not fully clear, but it is theorized that low oxygen tension results in fibrocartilaginous metaplasia, which leads to formation of the calcium deposit [1–3]. This condition differs from calcium deposition from degenerative tendinopathy [3] and is characterized by four stages of disease (pre‐calcific, formative, resorptive, and post‐calcific). Imaging modalities to diagnosis calcific tendinopathy include radiographs, CT scan, US, and MRI. The condition can cause severe pain and is managed symptomatically with oral pain medications (including anti‐inflammatories and opioids), subacromial steroid injections, and intra‐lesional steroid injections. More severe cases can be treated with extra‐corporeal shock wave therapy and with surgical excision [1–3].

Calcific tendinopathy has been reported in other appendicular structures and noted in imaging reviews [3, 4]. However, there is no significant documentation in the literature of calcific tendinopathy of the proximal hamstring origin [2, 3]. The hamstring muscle group is composed of three muscles with a total of four components, the semimembranosus, semitendinosus, and the biceps femoris [5–8]. The biceps femoris has a short and long head. The short head originates from the posterior femoral shaft. The long head of the biceps originates with the other two muscles from the ischial tuberosity. The semimembranosus typically originates on its own from the lateral facet of the ischial tuberosity while the long head of the biceps and the semitendinosus originate from the medial facet. Typically, proximal hamstring pathology involves degrees if tendon injury ranging from tendinosis, partial tear, or a complete tear [7]. These degrees of pathology are generally identified using MRI, but ultrasound can also be used. However, there is no gold standard identified to diagnose proximal hamstring calcific tendinopathy [3]. Similar to tendon pathology in other sites, hamstring pathology is generally treated with physical therapy, anti‐inflammatories, injections, or surgery.

Typically, open hamstring repair is done for high‐grade partial or full thickness tears of one of both components of the tendon origin [7, 8]. However, over the past 10–15 years, proximal hamstring endoscopy has emerged as an option to address proximal hamstring pathology [7, 9]. As in diagnostic endoscopy of other sites, this approach offers the potential to identify other pathology missed by diagnostic imaging and some studies show fewer wound and cutaneous nerve complications [9].We present two cases, with patient consent obtained, of proximal hamstring calcific tendinopathy identified through proximal hamstring endoscopy. Proximal hamstring endoscopy is a recent technology that can be used to identify and manage patients with proximal hamstring calcific tendinopathy.

## 2. Surgical Technique

**Figure 1:**
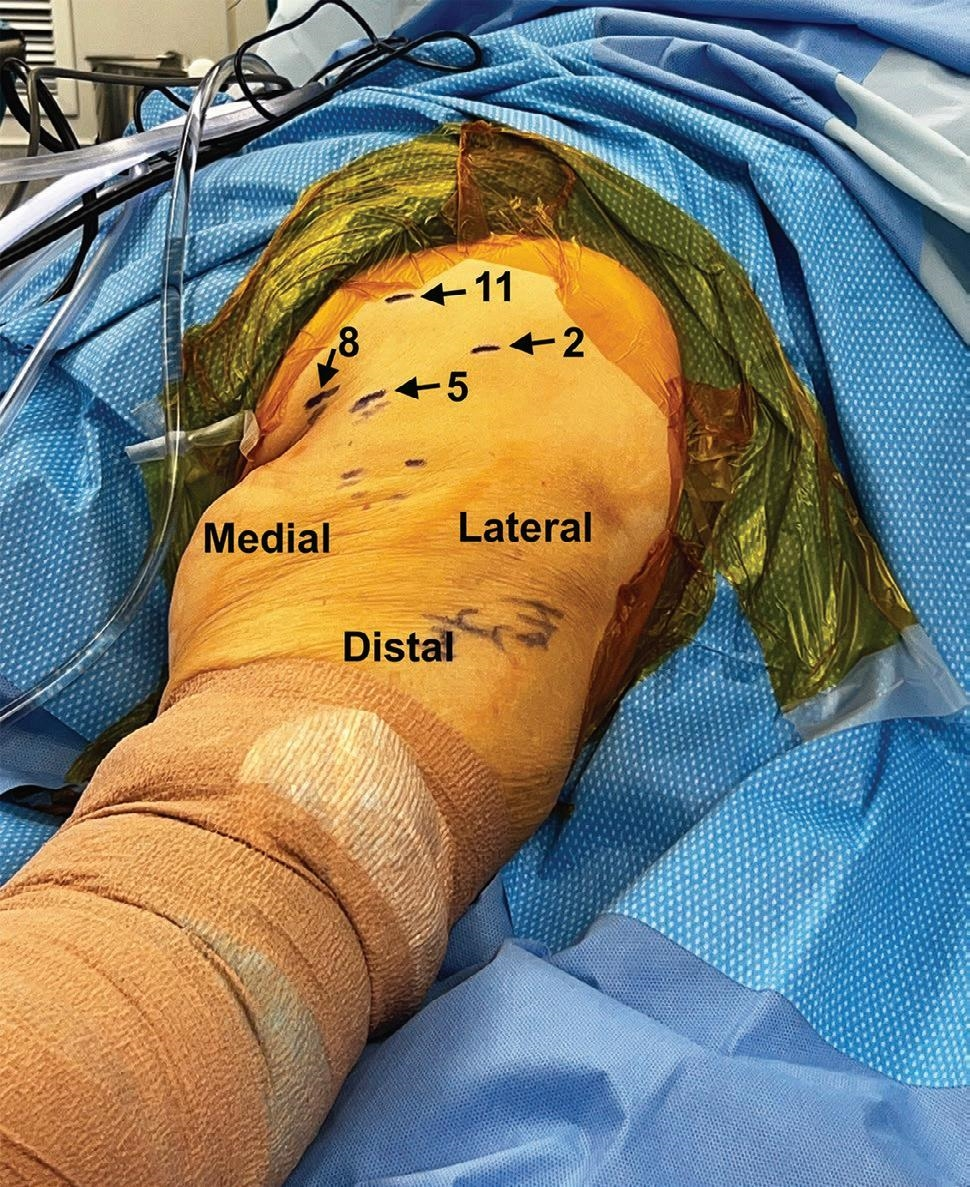
Portal sites marked for a right proximal hamstring endoscopy. The portal sites are placed approximately at the 2, 5, 8, and 11 o’clock positions. The 5 o’clock position is the typical camera viewing portal. Most surgical work is done through the 2 o’clock portal. The other two portals are mainly used for protection of the sciatic nerve or for suture management.

The senior surgeon uses an endoscopic technique, which does not require fluoroscopy. Both cases were done with the same steps. A configuration of four portals is placed sequentially at the 11, 2, 5, and 8 o’clock positions in a right hamstring as described below (Figure 1). A general outline of the surgical technique is presented here with individual cases to follow:

1. The patient is placed under general anesthesia and then positioned prone. All bony prominences are padded.
2. A very mild flex (20°) is placed in bed at the level of the iliac crests. The entire leg is prepped and draped to midline and to include the entire ileum in the field. A pillow is placed beneath the leg and under the drapes to keep the knee in mild flexion.
3. The distal (5 o’clock) portal is placed first. This is done by palpating the ischial tuberosity and making a transverse incision 1 cm in length approximately 3–5 cm distal to the gluteal crease depending on patient size. A clamp is used to spread the subcutaneous tissue. A standard, 4 mm 30° knee arthroscopy camera (Stryker, Kalamazoo, MI) trochar is inserted gently to palpate the ischial tuberosity. Gently sweeping is done along the tuberosity and the 30° knee arthroscopy camera is then inserted with the flow initiated at 30°40 mmHg.
4. The 2 o’clock portal incision is made similarly using a scalpel and spreading with a clamp. The blunt metal trochar is inserted to palpate the ischial tuberosity and triangulation as well as direct visualization with the camera are used to bluntly clear adventitial tissue and fascia to clear the surface and expose the ischial tuberosity. The sciatic nerve is also exposed and visualized in this step or in step 5.
5. Once visualization is adequate, the 2 o’clock portal is slight enlarged to allow placement of an 8‐mm diameter hip arthroscopy cannula (Stryker, Kalamazoo, MI). A curved 4.5 mm hip arthroscopy shaver (Stryker, Kalamazoo, MI) is then used to continue to improve visualization, but care is taken to avoid shaving deep and lateral to the ischial tuberosity to avoid injury to the sciatic nerve and the posterior femoral cutaneous nerve.
6. The 11 o’clock and 8 o’clock portals are created using spinal needles for direct visualization and are placed at distances to allow access without crowding the other portals.

## 3. Case Presentation

### 3.1. Case One

A 54‐year‐old female presented with progressive left hamstring pain 2 years after an injury using a treadmill. She had trouble sitting, walking, and standing. Conservative management included physical therapy and anti‐inflammatory medications. She also had a fluoroscopic guided cortisone injection 6‐weeks prior to the procedure, but it was not given in the ischial tuberosity, and a subsequent MRI demonstrated the same appearance as an MRI done 2 years prior. Her medical history was significant for a prior diagnosis of breast cancer and hypothyroidism.

On physical examination, she was tender over the left ischial tuberosity and had pain with resisted knee flexion and hip extension. MRI of the pelvis and proximal hamstring was performed using a 1.5‐T GE system with multiplanar, multisequence imaging, which demonstrated a 50% partial‐thickness undersurface tear of the semimembranosus tendon insertion of the proximal hamstring (Figure 2a,b). Given her persistent pain and functional limitations, despite conservative treatment, the patient proceeded with endoscopic repair.

Figure 2(a–b). Coronal and axial T2 weighted images for Case #1. The study was done on a GE 1.5 T machine. There is subtle undersurface signal of the semimembranosus insertion at the ischial tuberosity (see red circle). (a) Coronal, (b) Axial.

**Figure:**
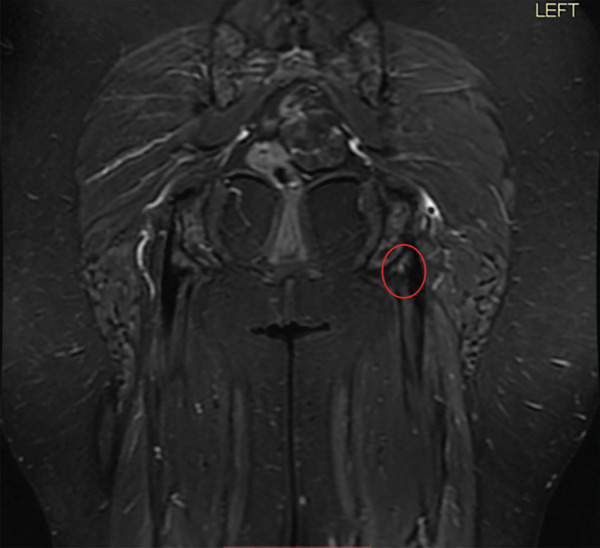
(a)

**Figure:**
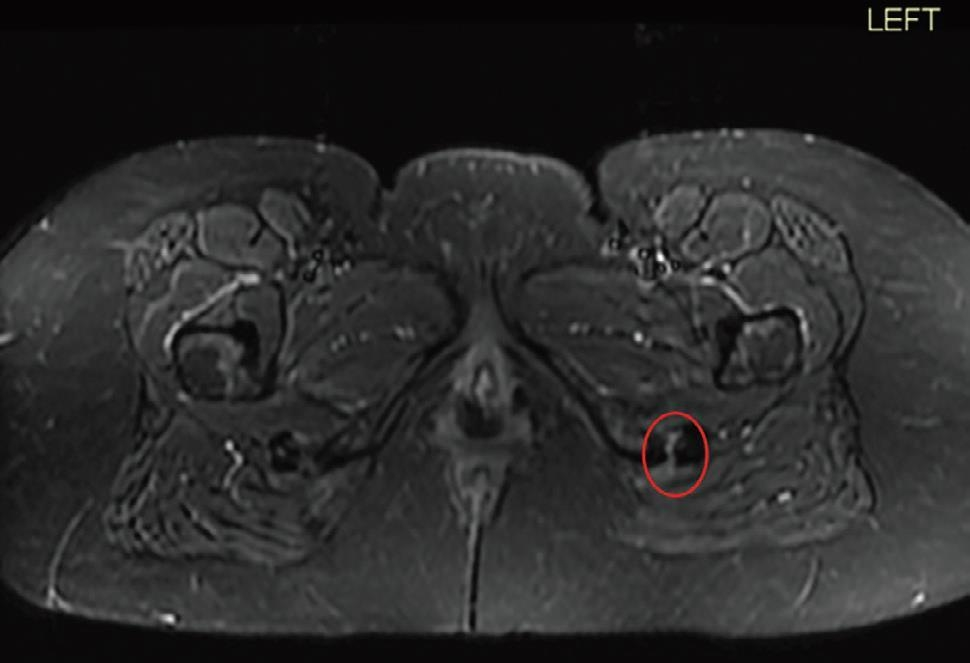
(b)

Four arthroscopic portals were used to access the proximal hamstring. Intraoperatively, the superficial surface of the semimembranosus tendon insertion was intact with visible calcific deposits embedded within the fibers (Figure 3a,b). A vertical incision was made in the tendon and there was efflux of copious chalky white material. The chalky material was debrided, and the surface of the ischium was prepared with a shaver. One 3.4‐mm PEEK anchor (Mitek, Warsaw, IN) was placed to repair the tendon to the surface of the ischial tuberosity.

Figure 3Endoscopic view of the left ischial tuberosity of Case #1 obtained using a 4‐mm, 30° arthroscope, demonstrating calcification on the surface and within the proximal hamstring. The camera is positioned in the 7 o’clock portal and oriented inferiorly toward 12 o’clock, while the shaver is introduced through the 10 o’clock portal. The calcification appears as a focal fullness with a thin overlying layer of tendon. The shaver head is palpating the superficial surface of the tendon insertion and shows efflux of the chalky calcification through the fascia in image (a), and shows calcific tissue covered by a thin layer of adventitial fascia in image (b). There is a fullness of the hamstring insertion due to the presence of the calcification.

**Figure:**
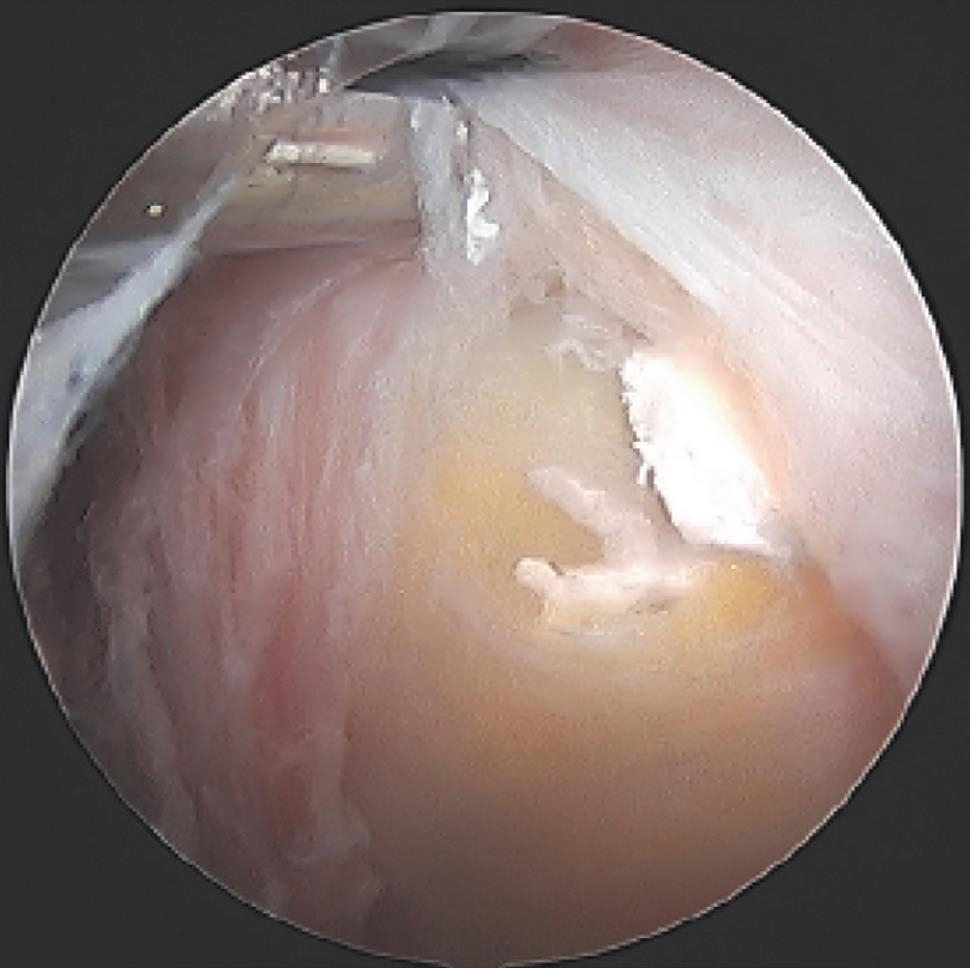
(a)

**Figure:**
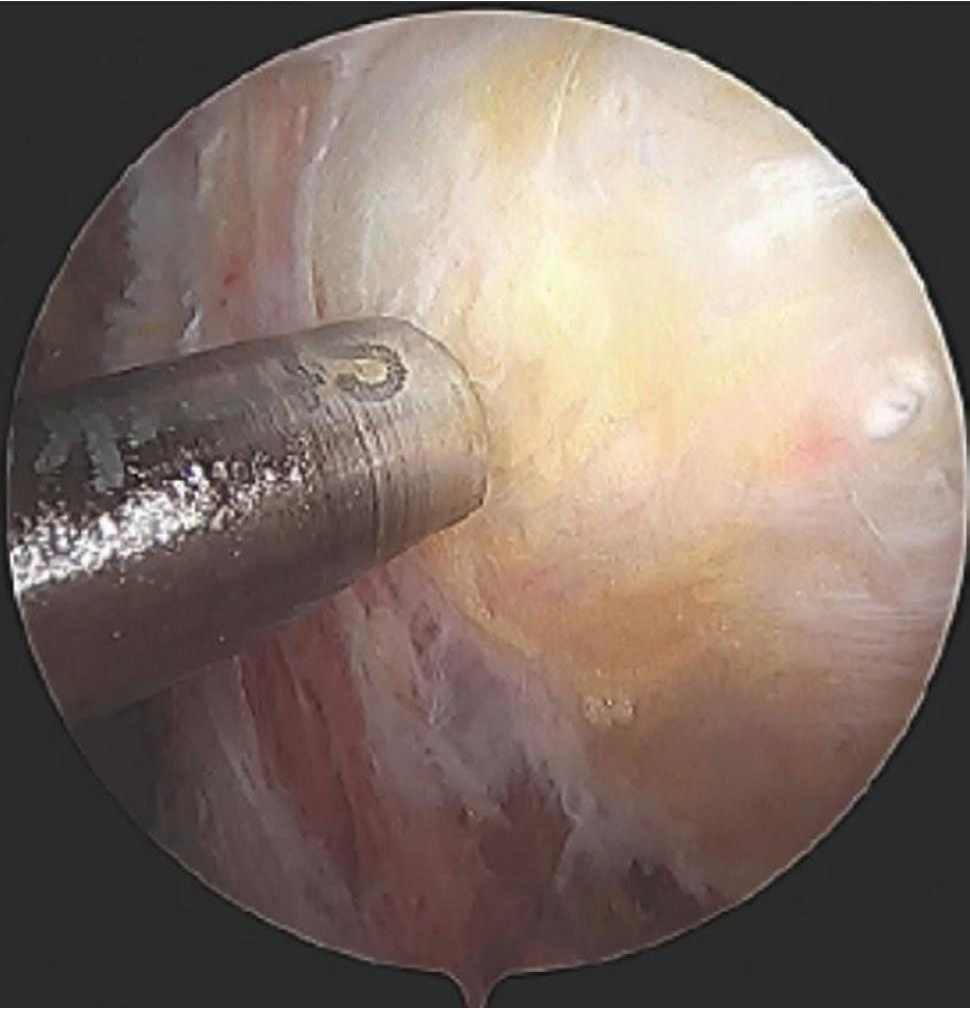
(b)

She stated at her first postoperative visit at 10 days that she had no pain. She continued to regain function at her 3‐month follow‐up and did not return for further follow‐up.

### 3.2. Case Two

A 72‐year‐old female presented with right hamstring pain that had been progressively worsening over 4 months. She described aching discomfort in the posterior thigh occasionally radiating to the knee, aggravated by prolonged standing, sitting, and walking. Her past medical history was significant for peripheral neuropathy, thyroid disease, chronic pain, and osteoporosis. She was a former smoker with a 20‐pack‐year history. She did not have any prior injections.

On physical examination, she was tender over the right ischial tuberosity and had pain with hip flexion with simultaneous knee extension. She walked with an antalgic gait. MRI of the pelvis was performed using a 3.0‐T GE system, with scans obtained in the sagittal, axial, and coronal planes utilizing T1‐weighted, spin‐density fat‐suppressed, and T2‐weighted pulse sequences, which demonstrated a 90% high‐grade partial‐thickness tear of the conjoined tendon of the proximal hamstring origin (Figure 4a,b). She also had a symptomatic right knee medial meniscus tear. She was under the care of pain management and desired surgical intervention for both. Preoperative patient‐reported outcome measures demonstrated a SANE score of 42, a VAS pain score of 74, a KOOS‐JR score of 59.4, and a PROMIS‐10 Physical Health score of 39.8. She underwent right knee arthroscopy with partial medial meniscectomy and proximal hamstring endoscopy.

Figure 4(a–b). Coronal and Axial T2 weighted images for Case #2. The study was done on a GE 3.0 T machine. There is significant fluid with near complete detachment of the undersurface attachment of the proximal hamstring tendon insertion at the ischial tuberosity (see red circle). (a) Coronal, (b) Axial.

**Figure:**
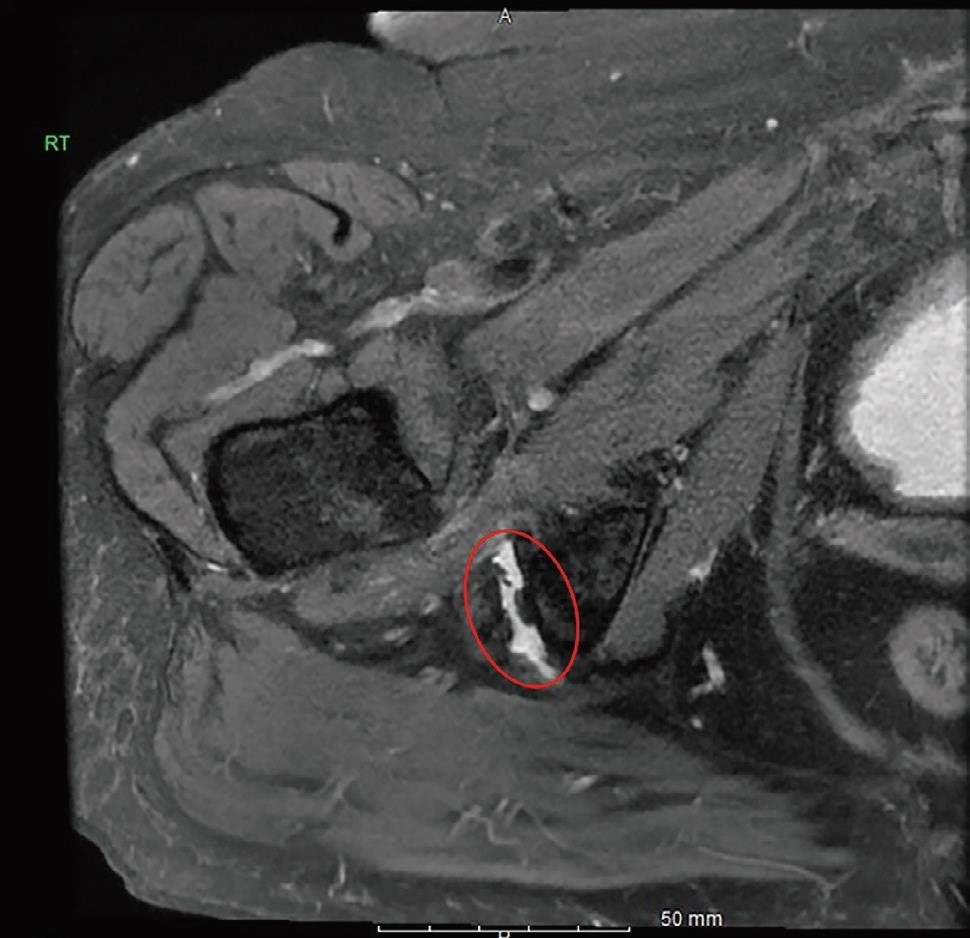
(a)

**Figure:**
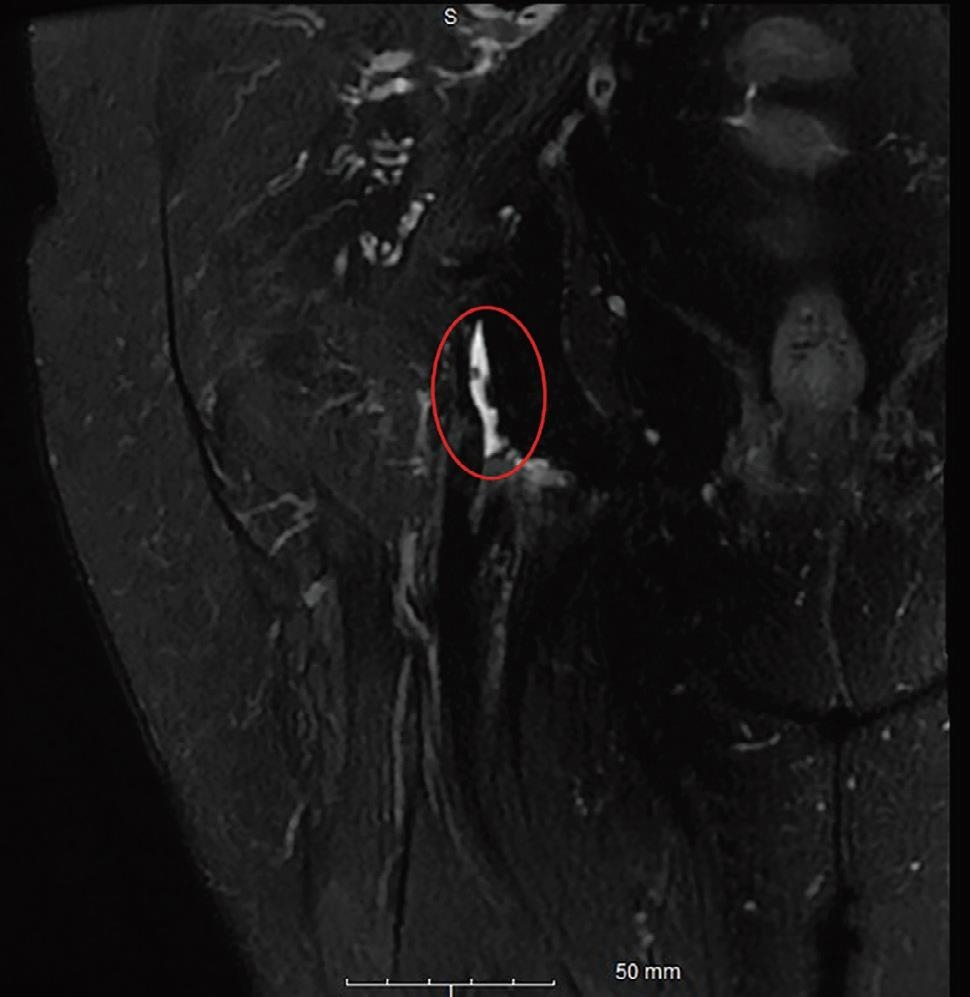
(b)

After completing the knee arthroscopy, the patient was placed in a prone position. Four arthroscopic portals were used. The surface of the conjoined tendon was noted to be intact (Figure 5a), but upon vertical entry with a blade, calcification extravasated from the hamstring origin (Figure 5b). The chalky white calcium was debrided, the tuberosity was abraded (Figure 5c), and two anchors (Stryker, Kalamazoo, MI) were placed for repair (Figure 5d). The deep undersurface tear was opposed to the ischium using the anchors.

Figure 5(a–d). Endoscopic view of the right ischial tuberosity of Case #2 using a 4‐mm, 30° arthroscope showing the fullness of the tendon attachment (a), efflux of calcification (b), post debridement (c), and final repair (d). The 30° camera is positioned in the 5‐o’clock portal facing toward 8 o’clock, with the cannula and instruments visualized from the 2‐o’clock portal and additional instruments seen from the 8‐ and 11‐o’clock portals (Figure 1).

**Figure:**
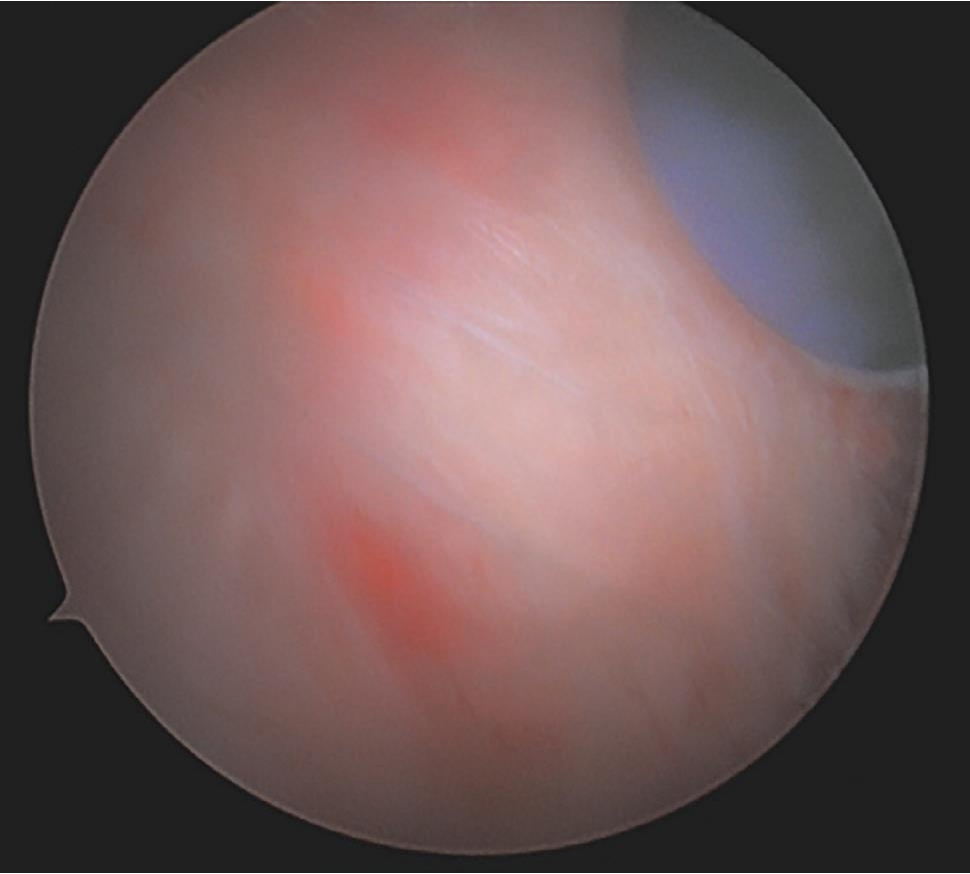
(a)

**Figure:**
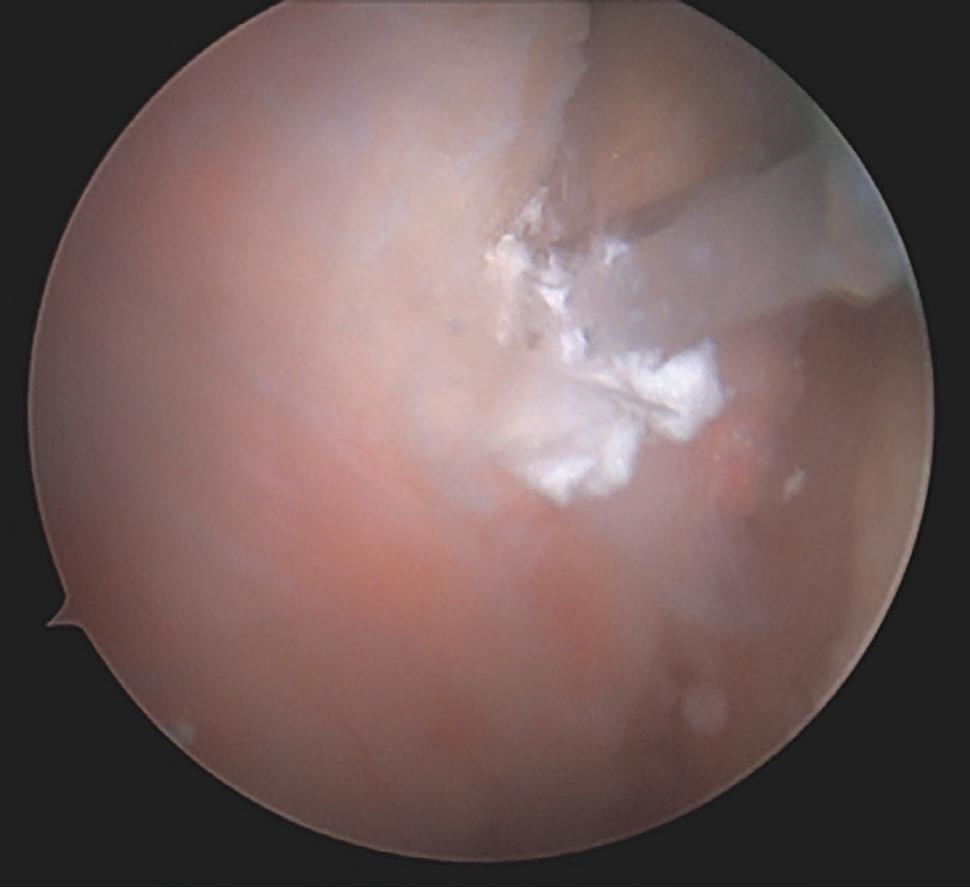
(b)

**Figure:**
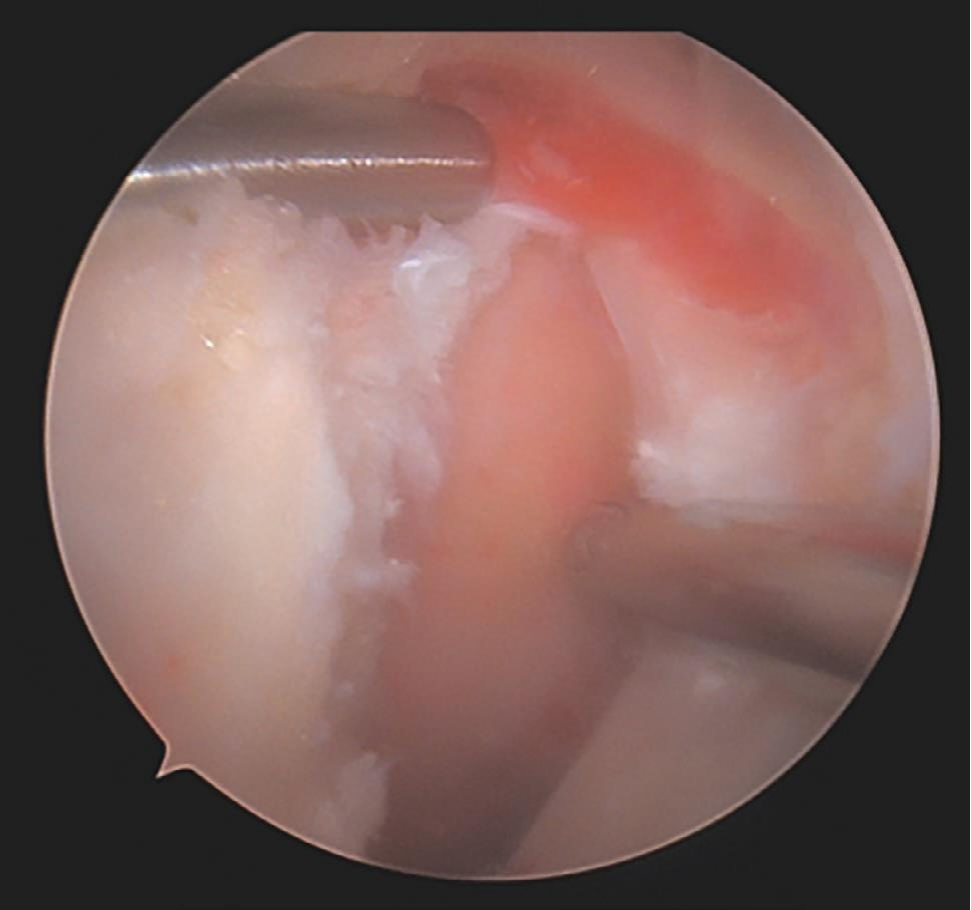
(c)

**Figure:**
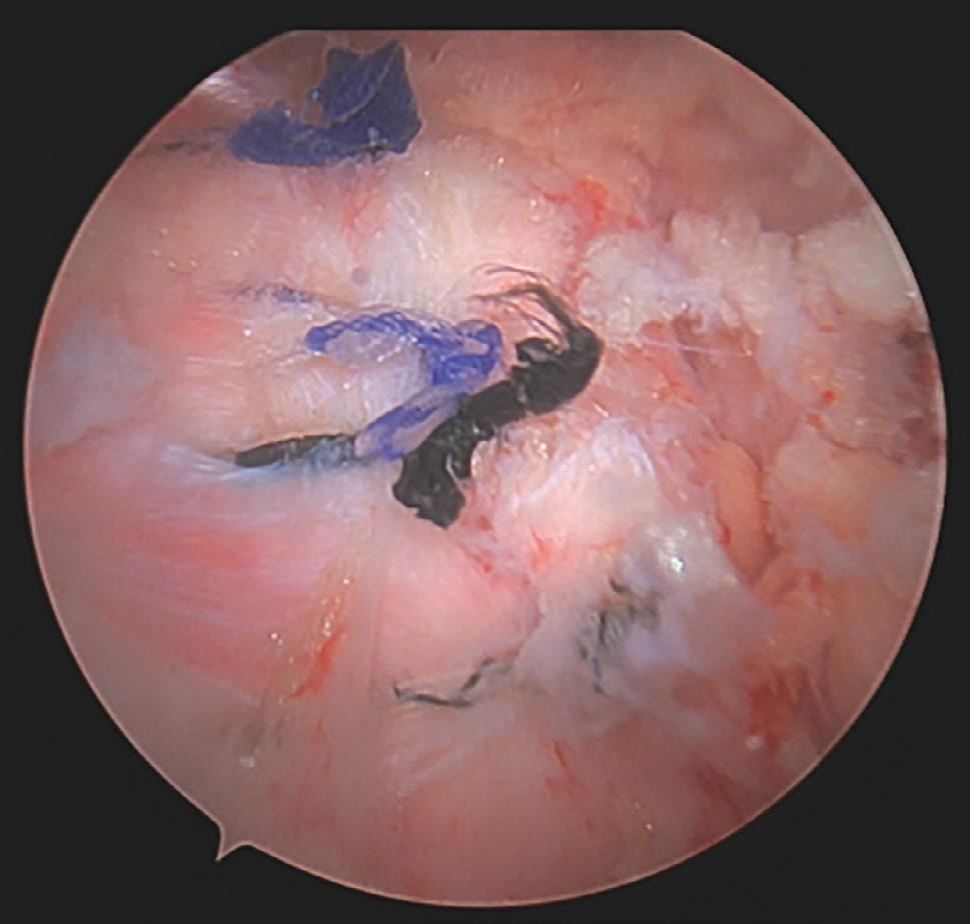
(d)

She reported immediate relief from the procedure at her first postoperative appointment and continued to improve at 2 months post‐surgery, sitting, walking, and standing with little pain. Postoperatively, patient‐reported outcomes at 3‐, 6‐, and 12‐months improved, with SANE scores of 90, 100, and 100; VAS pain scores of 30, 0, and 0; KOOS‐JR scores of 76.3, 100, and 100; and PROMIS‐10 Physical Health scores of 50.8, 50.8, and 54.1, respectively. At 1 year, the patient reported that the procedure “Far exceeded expectations,” was “Very satisfied with the outcome,” and “Would definitely choose to undergo the procedure again,” as assessed using three 5‐point Likert questionnaires on satisfaction.

## 4. Discussion

We present two patients diagnosed clinically and by MRI with partial tears of the proximal hamstring. Calcific tendinopathy was not a preoperative diagnosis, but proximal hamstring endoscopy allowed for visualization to make the diagnosis and facilitate debridement and repair of the tendon with low morbidity. The senior author has seen this diagnosis previously with open procedures, but the endoscopic approach allows for better visualization and less morbidity for the patient. This senior author has found that the endoscopic approach has advantages over an open approach because it is less invasive and less painful than the open approach, it reduces the risk of injury to the posterior femoral cutaneous nerve, it reduces the risk for wound infection or breakdown in a sensitive area, and in large patients, it provides better visualization and access to the hamstring insertion [9].

Although MRI remains the primary modality for evaluating proximal hamstring pathology, its sensitivity for detecting calcific tendinopathy may be limited. Ultrasound may be better suited for identifying calcific deposits due to its ability to detect echogenic foci with acoustic shadowing [3]. In these cases, MRI was valuable for confirming partial‐thickness tendon injury and excluding alternative diagnoses, which guided clinical decision‐making. The calcific tendinopathy was instead identified intraoperatively during endoscopic evaluation. These findings suggest ultrasound may serve as a complementary diagnostic tool in patients with refractory proximal hamstring pain, while MRI remains important for structural assessment.

Intraoperatively, the surface of the tendons appeared full, and, in one patient, the calcification was eroding through the surface of the tendon. Both cases required a longitudinal split in the tendon to perform debridement and repair.

These case reports illustrate the role of proximal hamstring arthroscopy to identify and treat calcific tendinopathy of the proximal hamstring. Calcific tendinopathy of the proximal hamstring is likely more common than is currently recognized and proximal hamstring endoscopy is a tool to consider when patients complain of moderate pain despite failure of conservative management of proximal hamstring pain. Both patients had relatively rapid relief of their pain and functional restoration.

## Funding

The Fondren Orthopedic Research Institute at the Texas Orthopedic Hospital supported a portion of the study team.

## Disclosure

The authors have nothing to report.

## Ethics Statement

This study was approved by the Texas Orthopedic Hospital’s IRB committee (TOH268e). IRB documentation is available upon request.

## Consent

Patient consent was obtained.

## Conflicts of Interest

The authors declare no conflicts of interest.

## Data Availability Statement

The data that support the findings of this study are available on request from the corresponding author. The data are not publicly available due to privacy or ethical restrictions.
